# Supplementary material for: IL-33 Inhibits TNF-α-Induced Osteoclastogenesis and Bone Resorption
Source: Int J Mol Sci. 2020 Feb 8;21(3):1130. doi: 10.3390/ijms21031130 (PMC7038169; doi:10.3390/ijms21031130)
Supplement: Supplementary file 1 [file ijms-21-01130-s001.pdf]

Supplementary Materials:

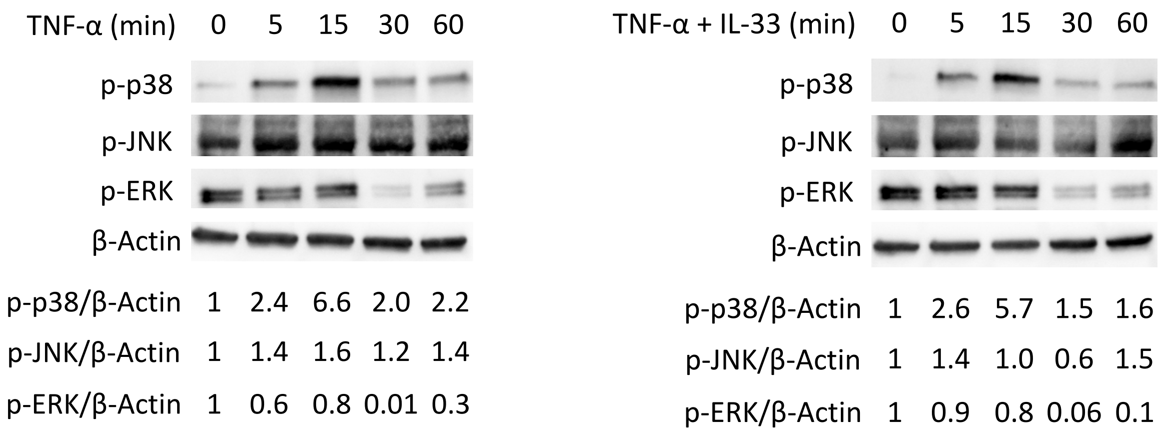

(**a**) (**b**)

Figure S1. IL-33 did not affect MAPK phosphorylation by TNF-α. Osteoclast precursors were exposed to (**a**) TNF-α or (**b**) TNF-α + IL-33 for specific periods. Cells were lysed and contents analyzed by western blotting.
